# Predictors of Smartphone and Tablet Use Among Patients With Hypertension: Secondary Analysis of Health Information National Trends Survey Data

**DOI:** 10.2196/33188

**Published:** 2022-01-24

**Authors:** Chinwe E Eze, Brady T West, Michael P Dorsch, Antoinette B Coe, Corey A Lester, Lorraine R Buis, Karen Farris

**Affiliations:** 1 College of Pharmacy University of Michigan Ann Arbor, MI United States; 2 Institute for Social Research University of Michigan Ann Arbor, MI United States; 3 Department of Family Medicine University of Michigan Ann Arbor, MI United States

**Keywords:** hypertension, mHealth, remote monitoring, telemonitoring, smartphones, tablets, text messaging, Health Information National Trends Survey, mobile health, digital health, mobile phone

## Abstract

**Background:**

Uncontrolled hypertension leads to significant morbidity and mortality. The use of mobile health technology, such as smartphones, for remote blood pressure (BP) monitoring has improved BP control. An increase in BP control is more significant when patients can remotely communicate with their health care providers through technologies and receive feedback. Little is known about the predictors of remote BP monitoring among hypertensive populations.

**Objective:**

The objective of this study is to quantify the predictors of smartphone and tablet use in achieving health goals and communicating with health care providers via SMS text messaging among hypertensive patients in the United States.

**Methods:**

This study was a cross-sectional, secondary analysis of the 2017 and 2018 Health Information National Trends Survey 5, cycles 1 and 2 data. A total of 3045 respondents answered “Yes” to the question “Has a doctor or other healthcare provider ever told you that you had high blood pressure or hypertension?”, which defined the subpopulation used in this study. We applied the Health Information National Trends Survey full sample weight to calculate the population estimates and 50 replicate weights to calculate the SEs of the estimates. We used design-adjusted descriptive statistics to describe the characteristics of respondents who are hypertensive based on relevant survey items. Design-adjusted multivariable logistic regression models were fitted to estimate predictors of *achieving health goals with the help of smartphone or tablet* and *sending or receiving an SMS text message to or from a health care provider in the last 12 months*.

**Results:**

An estimated 36.9%, SE 0.9% (183,285,150/497,278,883) of the weighted adult population in the United States had hypertension. The mean age of the hypertensive population was 58.3 (SE 0.48) years. Electronic communication with the doctor or doctor’s office through email or internet (odds ratio 2.93, 95% CI 1.85-4.63; *P*<.001) and having a wellness app (odds ratio 1.82, 95% CI 1.16-2.86; *P*=.02) were significant predictors of using SMS text message communication with a health care professional, adjusting for other demographic and technology-related variables. The odds of achieving health-related goals with the help of a tablet or smartphone declined significantly with older age (*P*<.001) and ownership of basic cellphones (*P*=.04). However, they increased significantly with being a woman (*P*=.045) or with being married (*P*=.03), having a wellness app (*P*<.001), using devices other than smartphones or tablets to monitor health (*P*=.008), making health treatment decisions (*P*=.048), and discussing with a provider (*P*=.02) with the help of a tablet or smartphone.

**Conclusions:**

Intervention measures accounting for age, gender, marital status, and the patient’s technology-related health behaviors are required to increase smartphone and tablet use in self-care and SMS text message communication with health care providers.

## Introduction

### Background

Among the 121.5 million adults in the United States with hypertension, 61.2% are aware of their disease condition, and 50.4% are receiving treatment, but only about 22% have their blood pressure (BP) controlled [1]. Uncontrolled hypertension can lead to stroke [2], systemic embolism and bleeding [3], congestive heart failure [4], myocardial infarction [5], renal damage, dementia, aortic aneurysm, angina pectoris, metabolic syndrome, diabetes, blindness, and death [6,7]. The 2021 Heart Disease and Stroke statistics report that 57.2% of all deaths recorded in the United States from 2008 to 2018 were attributed to hypertension [1]. Despite effective lifestyle and pharmaceutical treatments, the number of patients with uncontrolled BP in the United States is undesirable. Thus, there is a need to harness every possible arsenal to mitigate this challenge.

One strategy to improve BP control involves patients in their disease management through technology [8]. Recent innovations in information and communication technology provide excellent opportunities for improvements in hypertension control. There has been a steady increase in internet users and mobile cellular subscribers since 2000 [9]. According to a 2021 Pew Research Center report, 93% of adult Americans now use the internet, and an increase in internet use is seen across all age groups [9]. Moreover, 97% of adult Americans own a cellphone, and 85% now use smartphones [10].

In considering technology and BP control, patients with hypertension can now measure their BP using electronic monitors and transmit the results to their health provider through electronic health record platforms on their smartphones, tablets, or computers, and get feedback through the same channels without having to leave the comfort of their homes [11]. Phone calls, SMS text message alerts, health apps, emails, and alarms have also been used, and collectively this is called telemonitoring. Improvements in BP control have been noted with this type of remote monitoring. For example, a pharmacist-led telemonitoring intervention involving weekly electronic transmission of home-measured BP and regulated telephone visits among 450 patients with uncontrolled BP resulted in a significant decrease in systolic BP at 6, 12, and 18 months of −10.7 mm Hg (95% CI −14.3 to −7.3 mm Hg), *P*<.001; −9.7 mm Hg (95% CI −13.4 to −6.0 mm Hg), *P*<.001; and −6.6 mm Hg (−10.7 to −2.5 mm Hg), *P*=.004, respectively [11]. In addition, this study reported an increase in the proportion of patients with controlled BP in the telemonitoring group (71.8%, 95% CI 65.0-77.8) compared with the usual care group (57.1%, 95% CI 51.5-62.6) [12]. More generally, the use of SMS text messages as reminders and health education delivery led to improvements in behavior changes, hypertension knowledge, medication adherence, and BP among patients with hypertension [13-16]. A meta-analysis of 46 randomized controlled trials reported that home BP telemonitoring decreased systolic BP −3.99 mm Hg (95% CI −5.06 to −2.93; *P*<.001) and diastolic BP −1.99 mm Hg (95% CI −2.60 to −1.39; *P*<.001) in the intervention groups compared with usual care [17]. However, these are mostly intervention studies that are not nationally representative.

Although we know the advantages of these technologies in achieving favorable health outcomes, little is known about the predictors of their use among patients with hypertension. Using the Health Information National Trends Survey (HINTS), Langford et al [18] examined the prevalence of smartphones, basic phones, and tablets and compared respondents who are hypertensive and nonhypertensive. They found that 68%, 55%, and 16% of the hypertensive population had smartphones, tablets, and basic mobile phones, respectively. Younger respondents who are hypertensive were more likely to own a smartphone or tablet and have a health-related app. The ownership of smartphones or tablets increased with an increase in educational attainment. Another HINTS study focused on respondents with one or more chronic medical conditions and found that gender, age, employment status, and having a health app were associated with achieving a health-related goal with a smartphone or tablet. However, this study did not differentiate the respondents according to the disease conditions in the analysis [19]. Other studies on mobile health app use did not focus on people with hypertension [20,21]. Therefore, there is a need for more hypertension-focused studies to identify the factors that impact mobile health (mHealth) technology use among this patient population.

### Objectives

The aim of this study is to quantify the predictors of smartphone and tablet use in achieving health goals and communicating with health care providers via SMS text messaging among patients with hypertension. Our research question was, “What are the relationships of patients’ characteristics with the use of a smartphone or tablet to achieve health goals and sending or receiving text messages to or from healthcare professionals, among a nationally representative sample with hypertension?” This study provides nationally representative estimates regarding the predictors of using a smartphone or tablet to achieve health-related goals and SMS text messaging communication with health care professionals among respondents who are hypertensive. It also illuminates respondents’ factors associated with the use of these communication approaches. This will help us identify where and how to channel efforts to improve involvement of patients in telemonitoring of BP when health care providers work with their patients to increase smartphone and tablet use for health services. These results will also inform our questions for further studies to understand patients’ experiences with technology for BP control.

## Methods

### Design

This study was a cross-sectional, secondary quantitative analysis of the 2017 and 2018 HINTS 5, cycles 1 and 2 data. We combined the 2 cycles to provide more robust estimates of our relationships of interest. The study was considered exempt by the University of Michigan institutional review board (approval number: HUM00208364).

### Data Collection

The HINTS was developed by the Health Communication and Informatics Research branch of the National Cancer Institute. It is a publicly available, nationally representative survey that monitors how American adults aged ≥18 years obtain and use health information. HINTS has been carried out every few years since 2003, and the target population is adult Americans aged ≥18 years in the civilian noninstitutionalized population of the United States. HINTS uses a 2-stage sampling design, and residents in high minority strata are oversampled. A high minority stratum represents places with ≥34% Hispanic or African Americans. The data had both a full sample weight and 50 replicate weights assigned to each completed questionnaire for the adult sample. The 50 replicate weights were computed using the jackknife replication method. The full sample weight enables the calculation of population and subpopulation estimates, whereas the 50 replicate weights allow for the analysis of design-adjusted SEs for these estimates. The sample weights allow valid inferences from the responding sample to the population, accounting for unequal probability of selection, nonresponse, and noncoverage biases. The details of the sampling methods and weighting approaches are available in the HINTS 5, cycles 1 and 2 methodology reports [22,23].

### Participants or Sample Size

A total of 6789 respondents completed the HINTS 5 cycles 1 and 2 questionnaires. Respondents to HINTS who answered “*Yes*” to the question “Has a doctor or other health provider ever told you that you had high blood pressure or hypertension?” were the subpopulations used in this study. Out of the 6789 respondents, 3045 (44.85%) belonged to this subpopulation and thus constituted the final sample included in this analysis.

### Variables of Interest

The dependent variables were (1) Has your tablet or smartphone helped you track progress on a health-related goal, such as quitting smoking, losing weight, or increasing physical activity? (yes or no) and (2) Have you sent or received an SMS text message from a doctor or other health care professional within the last 12 months? (yes, no, or don’t know). The *no* and *don’t know* responses were combined to a single *no* response for logistic regression analysis. In this study, we described and predicted these variables. We selected these 2 items because they are most closely related to the concept of telemonitoring of BP. We also provided population proportion estimates of the following variables: Has your tablet or smartphone helped you make a decision about how to treat an illness or condition? (yes or no); Has your tablet or smartphone helped you in discussions with your health care provider? (yes or no); Other than a tablet or smartphone, have you used an electronic device to monitor or track your health within the last 12 months? (yes or no); Have you shared health information from either an electronic monitoring device or smartphone with a health professional within the last 12 months? (yes or no); and in the past 12 months, have you used a computer, smartphone, or other electronic means to use email or the internet to communicate with a doctor or doctor’s office? (yes or no).

The independent variables included respondents’ demographics (such as age, educational level, marital status, and income) and clinical characteristics (BMI, comorbidities, and general health status). Technology-related covariates included technology access, such as ownership of smartphones, tablets, wellness health apps, and basic cellphones. Technology-related behaviors, such as electronic communication with the doctor or doctor’s office through email or the internet were also included. These covariates were selected as they are technology-related items that can be applied to BP telemonitoring.

### Statistical Analysis

We accounted for the sampling weights and complex sample design features in all analyses to obtain population-level estimates for the United States using the R *survey* package by Thomas Lumley. Variance estimates were computed using the jackknife replication method, and specialized (unconditional) subpopulation analyses were not required when using this replication approach [24]. Descriptive statistics were used to analyze the characteristics of the respondents based on relevant demographics and covariates. We fit multivariable logistic regression models to the variables of interest to determine the most important predictors of the dependent variables. We first used demographic variables only and then tested the full model with clinical and technology use variables. The pseudo maximum likelihood estimation method was used to fit the regression models. To arrive at the final fitted model, we used a step-by-step approach starting from the preliminary bivariate analyses of potential predictors, followed by fitting different models containing all the anticipated predictors and variables of interest as well as interaction terms. We used the *regTermTest* function in the *survey* package to test the significance of the predictors using the design-adjusted Wald tests. None of the interaction terms was found to be significant. We identified the best-fitting model by choosing the model with the lowest design-adjusted Akaike information criterion [25]. Some nonsignificant predictors were retained in the models because they were found to be associated with hypertension in previous studies [26-28] and removing them did not result in a better-fitting model. Statistical significance was set at *P*≤.05. All analyses were conducted using the JJ Allaire R Studio (version 3.6.1).

## Results

### Demographics and Clinical Characteristics

Out of the 497,278,883 estimated weighted population surveyed, 183,285,150 (36.9%, SE 0.9%) responded “Yes” to having hypertension. The 183,285,150 estimated hypertensive population constituted the denominator for all analyses in this study. The mean age of the hypertensive population was 58.3 (SE 0.48) years. Among people with hypertension, there were more men (52.7%) than women (47.3%), and most persons were aged between 50 and 64 years (Table 1). The hypertensive population was predominantly non-Hispanic White people (66.9%), and most had some college education or more (61.2%). Most were married or living as married (57.1%), and more than three-quarters considered themselves to be in good, very good, or excellent health. Less than half of this subpopulation was employed (46.7%), and more than two-thirds earned a yearly household income below US $75,000. Diabetes was the most commonly reported comorbidity (33.7%).

**Table 1:** Design-adjusted estimates of demographics and clinical characteristics among the hypertensive population (sample size=3045; estimated population size=183,285,150).

Variable and category			Value		95% CI
Age (years), mean (SE)			58.3 (0.48)		57.31-59.21
**Age groups (years), % (SE)**					
	18-34	6 (1)		4.1-8.0	
	35-49	22.2 (1.5)		19.2-25.2	
	50-64	37.6 (1.4)		34.8-40.3	
	65-74	18.8 (0.6)		17.6-19.9	
	≥75	15.4 (0.6)		14.2-16.6	
**Gender, % (SE)**					
	Male	52.7 (1.3)		50.1-55.3	
	Female	47.3 (1.3)		44.7-49.9	
**Education level, % (SE)**					
	Less than high school	10.8 (1)		8.8-12.8	
	High school graduate	28 (1.4)		25.3-30.8	
	Some college	37.4 (1.4)		34.6-40.2	
	College graduate or more	23.8 (1.0)		21.9-25.6	
**Race or ethnicity, % (SE)**					
	Non-Hispanic White	66.9 (1.1)		64.7-69.1	
	Non-Hispanic Black or African American	13.9 (0.8)		12.4-15.4	
	Hispanic	12.8 (0.9)		11.1-14.5	
	Non-Hispanic Asian	3.2 (0.5)		2.2-4.2	
	Non-Hispanic other	3.1 (0.4)		2.4-3.9	
**Marital status, % (SE)**					
	Married	54.7 (1.2)		52.3-57.1	
	Living as married	2.4 (0.4)		1.6-3.2	
	Divorced	11.4 (0.6)		10.2-12.3	
	Widowed	9.2 (0.6)		8.0-10.4	
	Separated	1.6 (0.3)		1.0-2.7	
	Never married	20.7 (1.3)		18.1-23.4	
**Household yearly income (US $), % (SE)**					
	<20,000	21.1 (1.2)		18.7-23.5	
	20,000 to 35,000	13.2 (0.8)		11.6-14.8	
	35,000 to <50,000	15.2 (1.1)		13.0-17.3	
	50,000 to <75,000	19.4 (1.2)		17.0-21.9	
	≥75,000	31.1 (1.2)		28.6-33.5	
**Employment status, % (SE)**					
	Employed	46.7 (1.6)		46.7-49.7	
	Unemployed	53.3 (1.6)		50.1-56.4	
**Smoked at least 100 cigarettes, % (SE)**					
	Yes	44.7 (1.6)		41.6-47.7	
	No	55.3 (1.6)		52.3-58.4	
BMI, mean (SE)			31.1 (19.1)		30.7-31.4
**General health, % (SE)**					
	Excellent	5.5 (0.6)		4.4-6.6	
	Very good	28 (1.5)		25.0-30.9	
	Good	42 (1.5)		38.9-45.0	
	Fair	20.3 (1.3)		17.9-22.8	
	Poor	4.3 (0.6)		3.1-5.4	
**Diabetes, % (SE)**					
	Yes	33.7 (1.4)		31.0-36.4	
	No	66.3 (1.4)		63.6-69.0	
**Heart condition, % (SE)**					
	Yes	15.8 (1)		13.8-17.7	
	No	84.2 (1)		82.3-86.2	
**Depression, % (SE)**					
	Yes	27.8 (1.3)		25.4-30.3	
	No	72.2 (1.3)		69.7-74.6	

### Ownership and Use of Electronic Devices

In the hypertensive subpopulation, the distribution of ownership of electronic devices was as follows: smartphones (69.4%); tablets (54.7%); and basic cellphones (21.8%; Table 2). Almost three-quarters (74%) had accessed the internet; however, lower proportions used their smartphones or tablets to achieve health-related goals (36.1%) and sent or received SMS text messages to or from their health care professionals (30%). Only one-third (33.6%) of the hypertensive population communicated electronically with their doctor or doctor’s office through email or the internet.

**Table 2:** Design-adjusted proportions for ownership and use of mobile health (mHealth) electronic devices among the hypertensive population (sample size=3045; estimated population size=183,285,150).

Variable and category				Value, n (%)		SE (%)		95% CI (%)
**Technology access and use**								
	**Have only basic cellphone**							
		Yes	39,974,491 (21.81)		1.10		19.65-23.96	
		No	143,310,659 (78.19)		1.10		76.04-80.35	
	**Have smartphone**							
		Yes	127,108,252 (69.35)		1.36		66.69-72.01	
		No	56,176,898 (30.65)		1.36		28.00-33.31	
	**Have tablet**							
		Yes	100,183,663 (54.66)		1.44		51.84-57.48	
		No	83,101,487 (45.34)		1.44		42.52-48.16	
	**Use internet**							
		Yes	135,631,011 (74)		1.30		71.44-76.56	
		No	47,654,139 (26)		1.30		23.44-28.56	
	**Have health apps**							
		Yes	74,395,442 (40.59)		1.76		37.15-44.04	
		No	108,889,708 (59.41)		1.76		55.96-62.85	
**Technology-related health behaviors**								
	**Make health treatment decision with mHealth**							
		Yes	62,738,507 (34.23)		1.97		30.36-38.09	
		No	120,546,643 (65.77)		1.97		61.91-69.64	
	**Discuss with health provider with help of tablet or smartphone**							
		Yes	61,730,439 (33.68)		1.51		30.72-36.64	
		No	121,554,711 (66.32)		1.51		63.36-69.28	
	**Used other devices apart from tablet and smartphone to monitor or track health**							
		Yes	76,154,980 (41.55)		1.25		39.10-44.01	
		No	107,130,170 (58.45)		1.25		55.99-60.90	
	**Shared health information from electronic device, tablet, or smartphone with health provider**							
		Yes	38,838,123 (21.19)		1.02		19.19-23.19	
		No	129,875,857 (70.86)		1.42		68.07-73.64	
		Not applicable	14,571,170 (7.95)		0.88		6.23-9.68	
	**Electronic communication with doctor or doctor’s office via email or internet**							
		Yes	61,620,467 (33.62)		1.33		31.02-36.22	
		No	121,664,683 (66.38)		1.33		63.78-68.98	
**Dependent variables**								
	**Achieve health goal with tablet or smartphone**							
		Yes	66,165,939 (36.1)		1.60		32.96-39.25	
		No	117,119,211 (63.9)		1.60		60.75-67.04	
	**Sent or received an SMS text message from the doctor**							
		Yes	55,022,202 (30.02)		1.40		27.27-32.78	
		No	128,262,948 (69.98)		1.40		67.22-72.73	

### Use of Tablet or Smartphone to Achieve Health-Related Goals

**Figure 1:**
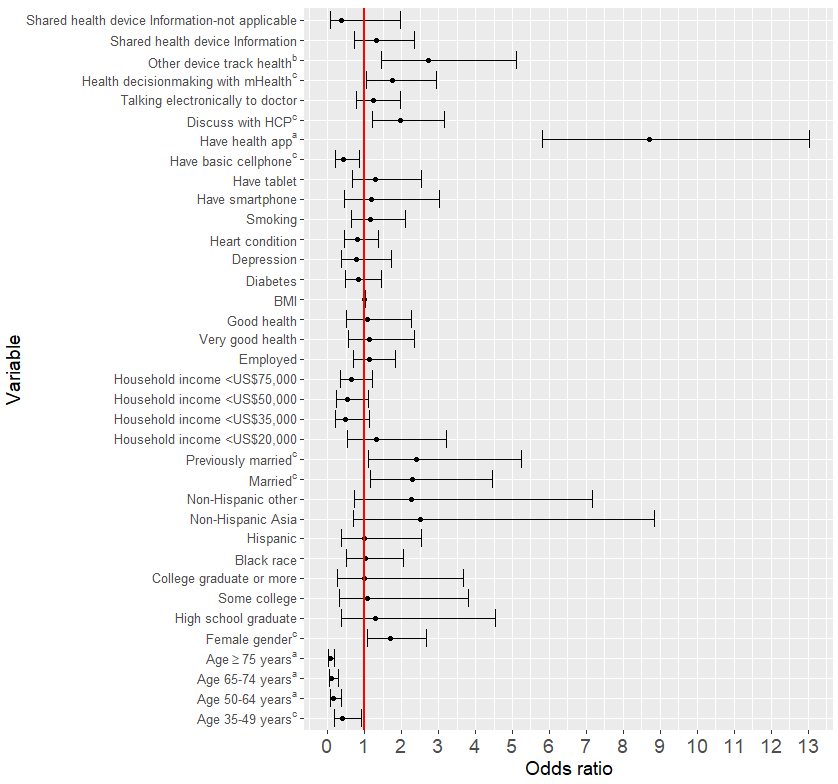
Full model with design-adjusted estimates of odds ratios for achieving health-related goal with the help of a tablet or smartphone among the hypertensive population (*P* values: ^a^.001, ^b^.01, ^c^.05). HCP: health care provider; mHealth: mobile health.

In the full model predicting *achieving health-related goals with the help of tablet or smartphone*, age, gender, marital status, ownership of basic cellphones, having a health-related wellness app, making health treatment decisions with mHealth, using devices other than tablets or smartphones to monitor or track health, and having a discussion with health care provider with the help of tablet or smartphone were significant predictors (Figure 1). In terms of the impact on the odds of achieving health-related goals with the help of tablet or smartphone, increasing age decreased the odds (35-49 years, odds ratio [OR] 0.41, 95% CI 0.18-0.91; 50-64 years, OR 0.17, 95% CI 0.08-0.38; 65-74 years, OR 0.11, 95% CI 0.04-0.29; >75 years, OR 0.07, 95% CI 0.02-0.19), being a woman increased the odds (OR 1.69, 95% CI 1.06-2.68), being married (OR 2.28, 95% CI 1.17-4.47) or previously married (OR 2.39, 95% CI 1.09-5.25) increased the odds, having a basic cellphone (OR 0.43, 95% CI 0.21-0.87) decreased the odds, having a wellness app (OR 8.70, 95% CI 5.81-13.04) increased the odds, making health decisions with mHealth (OR 1.77, 95% CI 1.06-2.94) increased the odds, tracking health with other devices (OR 2.73, 95% CI 1.46-5.12) and having discussion with the provider (OR 1.96, 95% CI 1.22-3.17) using tablet or smartphone increased the odds. Age, female gender, being married or previously married were also significant predictors of achieving health-related goals with the help of a tablet or smartphone when we accounted for only demographic variables (Multimedia Appendix 1). The reference categories for categorical predictors showed in Figure 1 include: age, 18-34 years; gender, male; education level, less than high school; race or ethnicity, non-Hispanic White; marital status, never married; household income, ≥US $75,000; employment status, unemployed; smoking, yes response; health status, fair; and other variables, no response. Also note the full expansion of the abbreviated variables in the figure as follows: Shared health device information: shared health information from electronic devices, smartphones, or tablets with health care providers; Other device track health: used other devices apart from tablet or smartphone to track health; Discuss with HCP: discuss with health care providers with the help of tablets or smartphones.

### Use of Tablet or Smartphone to Communicate With Health Care Provider Through Text Messaging

In the full model predicting *send or receive SMS text messages to or from a health care professional in the last 12 months*, electronic communication with the doctor or doctor’s office via email or internet (OR 2.93, 95% CI 1.85-4.63) and having health-related wellness apps (OR 1.82, 95% CI 1.16-2.86) were the only significant predictor variables (Table 3). Individuals who used a computer, smartphone, or other electronic means to use email or the internet to communicate with a doctor or doctor’s office in the past 12 months had 193% higher odds of sending or receiving SMS text messages from a health care professional in the last 12 months than those who did not. Those with health-related wellness apps had 82% higher odds of sending or receiving text messages from a health care professional in the last 12 months than those who did not. No other covariates were found to be statistically significant.

**Table 3:** Full model with design-adjusted estimates of odds ratios for sending or receiving text message from health care provider in the last 12 months among the hypertensive population (sample size=3045; estimated population size=183,285,150).

Predictor and category			Odds ratio (95% CI)		SE		*P* value
**Age group^a^ (years)**							
	35-49	2.22 (0.75-6.58)		0.55		.17	
	50-64	1.56 (0.54-4.53)		0.55		.43	
	65-74	1.48 (0.45-4.84)		0.61		.53	
	≥75	1.55 (0.43-5.59)		0.66		.52	
**Gender^b^**							
	Female	1.20 (0.83-1.74)		0.19		.36	
**Education level^c^**							
	High school graduate	0.84 (0.37-1.87)		0.41		.67	
	Some college	0.68 (0.30-1.52)		0.42		.36	
	College graduate or more	0.61 (0.24-1.54)		0.47		.31	
**Race or ethnicity^d^**							
	Non-Hispanic Black or African American	0.75 (0.43-1.31)		0.29		.33	
	Hispanic	0.90 (0.47-1.74)		0.34		.77	
	Non-Hispanic Asian	0.67 (0.23-2.06)		0.57		.495	
	Non-Hispanic other	1.57 (0.37-6.74)		0.74		.55	
**Marital status^e^**							
	Married	1.37 (0.68-2.77)		0.36		.39	
	Previously married	1.54 (0.65-3.65)		0.44		.35	
**Household yearly income^f^ (US $)**							
	<20,000	0.50 (0.22-1.15)		0.42		.13	
	20,000 to <35,000	0.55 (0.31-0.98)		0.29		.06	
	35,000 to <50,000	0.63 (0.34-1.16)		0.31		.16	
	50,000 to <75,000	0.82 (0.54-1.25)		0.22		.37	
**Employment status^g^**							
	Employed	0.80 (0.44-1.43)		0.30		.46	
**Smoked at least 100 cigarettes^h^**							
	No	1.21 (0.87-1.68)		0.17		.28	
**Health status^i^**							
	Very good	1.17 (0.66-2.09)		0.29		.60	
	Good	1.28 (0.79-2.09)		0.25		.33	
BMI			1.00 (0.96-1.03)		0.02		.78
**Diabetes^j^**							
	Yes	1.05 (0.69-1.60)		0.21		.82	
**Heart condition^j^**							
	Yes	1.04 (0.66-1.63)		0.23		.88	
**Depression^j^**							
	Yes	1.19 (0.70-2.05)		0.28		.53	
**Have smartphone^j^**							
	Yes	1.53 (0.62-3.80)		0.46		.37	
**Have tablet^j^**							
	Yes	1.08 (0.68-1.74)		0.24		.75	
**Have basic cellphone^j^**							
	Yes	0.79 (0.35-1.77)		0.41		.57	
**Have health apps^j^**							
	Yes	1.82 (1.16-2.86)		0.23		.02	
**Make treatment decision with mobile health^j^**							
	Yes	1.31 (0.84-2.04)		0.23		.26	
**Discuss with health provider with help of tablet or smartphone^j^**							
	Yes	0.98 (0.64-1.51)		0.22		.94	
**Used other devices apart from tablet and smartphone to monitor or track health^j^**							
	Yes	1.20 (0.79-1.84)		0.22		.40	
**Shared health info from electronic device, tablet, or smartphone with health provider^j^**							
	Yes	1.62 (1.02-2.56)		0.23		.06	
	Not applicable	0.74 (0.22-2.49)		0.62		.64	
**Electronic communication with doctor or doctor’s office via email or internet^j^**							
	Yes	2.93 (1.85-4.63)		0.23		<.001	

^a^Reference category: 18 to 34 years.

^b^Reference category: Male.

^c^Reference category: Less than high school.

^d^Reference category: Non-Hispanic White.

^e^Reference category: Never married.

^f^Reference category: ≥US $75,000.

^g^Reference category: Unemployed.

^h^Reference category: Yes response.

^i^Reference category: Fair.

^j^Reference category: No response.

Notably, in the model with only demographics, annual household income was the only significant predictor of sending or receiving SMS text messages to or from a health care professional in the last 12 months (Multimedia Appendix 2). Compared with the subpopulation with yearly household income of ≥US $75,000, the odds of sending or receiving SMS text messages from health care professionals in the last 12 months decreased by 40.0%, 50.7%, 64.7%, and 74.0%, respectively, among those with US $50,000 to <US $75,000 (OR 0.60, 95% CI 0.41-0.87; *P*=.01); US $35,000 to <US $50,000 (OR 0.49, 95% CI 0.28-0.88; *P*=.03); US $20,000 to <US $35,000 (OR 0.35, 95% CI 0.20-0.61; *P*=.001); and <US $20,000 (OR 0.26, 95% CI 0.13-0.51; *P*<.001) household incomes. The design-adjusted Wald test indicated that household income remained a significant predictor of sending or receiving SMS text messages to or from a health care professional (*F*_4,23_=4.92, *P*=.005).

## Discussion

### Principal Findings and Implications

The purpose of this study was to identify predictors of *using a smartphone or tablet to achieve health goals* and SMS *text messaging communication with health care professionals* among individuals with hypertension. Most of the hypertensive population have a smartphone, and just over half have tablets. We found that the likelihood of using a smartphone or tablet to achieve health-related goals significantly decreased with increase in age and ownership of a basic cellphone. The use of smartphones or tablets to achieve health-related goals was, however, statistically significantly positively associated with being a woman, being married or previously married, having a health-related wellness app, making health treatment decisions with mHealth, using devices other than tablets or smartphones to monitor or track health, and having a discussion with a health care provider with the help of a tablet or smartphone. Sending or receiving SMS text messages to or from a health care provider was statistically significantly positively associated with previous electronic communication with the doctor or doctor’s office by email or internet and having a health-related wellness app.

Achieving health-related goals with the help of a tablet or smartphone usually involves having a health-related app installed on a smartphone or tablet [29]. Therefore, it is not surprising that age was a significant predictor of achieving health-related goals with the help of a tablet or smartphone, with the odds decreasing as age increases. This may be because younger people are more likely to have smartphones, tablets, and health-related apps [10,18]. Studies have shown that older adults can use technology if they understand the benefits that they can get from such use [30-32]. Health care providers can recommend that older patients use their smartphones and tablets to achieve health goals and encourage more use. Our findings among people with hypertension in younger age and female gender as significant predictors of achieving health-related goals with the help of a tablet or smartphone agree with another HINTS study [19] among respondents with one or more chronic diseases, which found that respondents aged 65 years had lower odds compared with those aged between 18 and 34 years, whereas women had higher odds compared with men for tracking the progress of health-related goals with their tablet or smartphone. They also found that those with health-related apps have higher odds of tracking the progress of health-related goals with their tablet or smartphone than those who do not have the app, which agrees with our findings as well. However, their findings showed that being employed increases the odds and having good health status decreases the odds of tracking the progress of health-related goals with tablets or smartphones, which differ from ours, where employment and health status were not associated with tracking health goals. The difference in results could be because of the differences in the variables included in the regression models, or it could also be because their study was conducted among respondents with one or more chronic diseases. Another HINTS study [20] on adult respondents also found that the likelihood of achieving health goals with the help of the mHealth app decreased with increasing age.

It was not surprising that those who own only basic cellphones had lower odds of achieving health-related goals with the help of a tablet or smartphone compared with those who did not, because basic cellphones are not usually equipped with advanced features to do that. The significance of making health treatment decisions with mHealth, using devices other than a tablet or smartphone to monitor or track health and having a discussion with health care providers with the help of a tablet or smartphone as predictors for achieving health-related goals with the help of a tablet or smartphone buttresses the fact that people who are already using a technology device are more likely to increase their use than those who are not using a technology device. This finding suggests that these groups of the hypertensive population can also benefit from telemonitoring of BP. Health care providers can play a role in creating awareness of these resources and their usefulness among their patients. It is also critical that payment reform adequately recognizes providers’ time in supporting the telemonitoring of BP. The number of patients with hypertension using any of these technology devices could be increased by making them more affordable and accessible, and insurance coverage of such technology is likely necessary for the widespread adoption of telemonitoring of BP.

Less than one-third of the hypertensive subpopulation sent or received SMS text messages from their health care professionals. Interestingly, annual household income was a significant predictor of sending or receiving SMS text messages from a health care professional while considering only demographic variables, with the odds of sending or receiving SMS text messages decreasing with lower household income. SMS text messaging has been portrayed as a low-cost and common resource that can be used to improve health care. It has been shown to be effective in several intervention studies to improve hypertension knowledge and behavior changes, such as medication adherence and BP monitoring, leading to better BP control [13-16]. In general, 2-way SMS text messaging communication initiated by the health care provider keeps the patient and provider in frequent communication and is more effective in BP target attainment [14]. Our results show that the advantages of SMS text messaging are not being fully used in everyday life. One would think that SMS text messaging will be widespread across all income levels as it is considered an inexpensive option, but that is not the case. Advocacy for free SMS text messaging phone subscriptions for lower income patients with hypertension may increase the use of this technology. It could also be that patients are not aware that they can communicate with their health care professionals through SMS text messaging or that the service is not offered by their health providers. With adequate reimbursement, it behooves health care providers to initiate SMS text messaging with their patients so that they can both reap its advantages and free office time, to some extent, for more acute or serious visits.

In the full model, electronic communication with the doctor or doctor’s office and having a health-related wellness app were significant predictors of sending or receiving SMS text messages from a health care professional when we accounted for all covariates. This shows that those already in communication with their health care providers are more likely to continue even if there is a change in the communication channel. The significance of having a health-related wellness app suggests that those who are already doing a form of self-monitoring are more likely to communicate with their health care provider via SMS text messaging. These findings are important because the impact of demographic characteristics such as age, gender, and income was not statistically significant. This suggests that all individuals, not just the young or those with higher incomes, for example, should be targeted to use remote BP monitoring. An essential first step may be the first electronic communication with the doctor or doctor’s office, including email, electronic health portal messaging, or phone SMS text messaging. Having a health-related wellness app predicted both using a tablet or smartphone to achieve health goals and communication with a health care provider through SMS text messaging. These findings underscore the importance of these technology apps in improving health and the importance of the willingness of the patients to be more involved in their care through technology use. Health care systems could offer user-friendly health-related wellness apps to patients on a secure platform to boost patient trust and increase uptake.

### Study Limitations

Our study results are limited by the cross-sectional nature of the data, and the subpopulation used is based on self-reported hypertension. However, our robust analytic approach, which accounts for the HINTS sampling design, is positive. We also may not have accounted for all factors needed to predict the dependent variables because we used secondary data.

### Future Studies

Effective engagement with health technology requires patients to have some eHealth literacy [33]. eHealth literacy expresses a person’s understanding of the knowledge, skills, and resources needed to properly use health technology services. Future research should consider how factors, such as the eHealth literacy status of the patients or health care resources available to the patients are associated with the use of tablets or smartphones to achieve health goals and communicate with a health care provider through SMS text messaging in patients with hypertension. For example, mobile SMS text messages aimed at controlling child dental caries among parents with low eHealth literacy led to improvements in health outcomes and an increase in parental eHealth literacy in the intervention group at 6 months [34]. Further studies are required to understand how these predictors correlate with objective BP control and patient-provider communication preferences among patients with hypertension.

### Conclusions

The use of mHealth to achieve health goals and communicate with health care professionals by patients with hypertension is significantly associated with having health-related wellness apps. Achieving health goals is also associated with demographics, such as age, gender, marital status, technology access, and other technology-related behaviors. Communication with health care providers through SMS text messaging is associated with previous electronic communications with the doctor or doctor’s office. It is essential to consider these factors in tandem when planning telemonitoring for patients with hypertension. Measures accounting for these factors are required to increase smartphone and tablet use and their benefits in the routine care of patients with hypertension.

## Appendix

Multimedia Appendix 1 Demographics-only model with design-adjusted estimates of odds ratios for achieving health-related goals with the help of tablets or smartphones among the hypertensive population.

Multimedia Appendix 2 Demographics-only model with design-adjusted estimates of odds ratios for sending or receiving text messages from health care providers in the last 12 months among the hypertensive population.

## Abbreviations

BP: blood pressure
HINTS: Health Information National Trends Survey
mHealth: mobile health
OR: odds ratio
