# Supplementary material for: Predictors of Smartphone and Tablet Use Among Patients With Hypertension: Secondary Analysis of Health Information National Trends Survey Data
Source: J Med Internet Res. 2022 Jan 24;24(1):e33188. doi: 10.2196/33188 (PMC8822436; doi:10.2196/33188)
Supplement: Multimedia Appendix 1 [file jmir_v24i1e33188_app1.docx]

(sample size = 3045; estimated population size = 183,285,150)

| Predictor and category | | Odds ratio (95% CI) | SE | *P*-value |
| --- | --- | --- | --- | --- |
|  |  |  |  |  |
| **Age group**^a^ **(years)** | | | | |
|  | 35-49 | 0.36 (0.155-0.831) | 0.428 | .03 |
|  | 50-64 | 0.14 (0.058-0.353) | 0.462 | ˂.001 |
|  | 65-74 | 0.10 (0.037-0.270) | 0.506 | ˂.001 |
|  | ≥75 | 0.07 (0.024-0.180) | 0.514 | ˂.001 |
| **Gender**^b^ | | | | |
|  | Female | 1.69 (1.198-2.371) | 0.174 | .01 |
|  |  |  |  |  |
| **Education level**^c^ | | | | |
|  | High School graduate | 1.38 (0.611-3.115) | 0.415 | .45 |
|  | Some college | 1.80 (0.789-4.104) | 0.420 | .18 |
|  | College graduate or more | 1.75 (0.736-4.169) | 0.443 | .22 |
| **Race/ethnicity**^d^ | | | | |
|  | Non-Hispanic Black or African American | 1.27 (0.762-2.115) | 0.260 | .37 |
|  | Hispanic | 0.75 (0.366-1.526) | 0.364 | .43 |
|  | Non-Hispanic Asian | 2.17 (0.840-5.592) | 0.484 | .12 |
|  | Non-Hispanic other | 1.68 (0.626-4.483) | 0.502 | .32 |
| **Marital status**^e^ | | | | |
|  | Married | 2.41 (1.413-4.120) | 0.273 | .004 |
|  | Previously married | 2.11 (1.178-3.783) | 0.298 | .02 |
| **House-hold yearly income**^f^ **(US$)** | | | | |
|  | <20,000 | 0.57 (0.292-1.107) | 0.340 | .11 |
|  | 20,000 to <35,000 | 0.39 (0.212-0.722) | 0.312 | .01 |
|  | 35,000 to <50,000 | 0.50 (0.255-0.990) | 0.345 | .058 |
|  | 50,000 to <75,000 | 0.60 (0.382-0.936) | 0.228 | .03 |
| **Employment status**^g^ | | | | |
|  | Employed | 1.17 (0.804-1.715) | 0.193 | .41 |
| **Smoked at least 100 cigarettes**^h^ | | | | |
|  | No | 1.10 (0.711-1.716) | 0.223 | .66 |
| **Health status**^i^ | | | | |
|  | Very good | 1.07 (0.620-1.859) | 0.280 | .80 |
|  | Good | 0.93 (0.528-1.643) | 0.290 | .81 |
| **BMI** | | | | |
|  |  | 1.01 (0.985-1.037) | 0.013 | .43 |
| **Diabetes**^j^ | | | | |
|  | Yes | 1.07 (0.701-1.623) | 0.214 | .77 |
| **Heart condition**^j^ | | | | |
|  | Yes | 1.05 (0.523-2.106) | 0.355 | .89 |
| **Depression**^j^ | | | | |
|  | Yes | 0.95 (0.662-1.361) | 0.183 | .78 |

^a-j^Reference categories for categorical predictors.

^a^ =18 to 34 years; ^b^ =Male; ^c^ =Less than high school; ^d^ =Non-Hispanic White; ^e^ =Never married; ^f^ = ≥US$75,000; ^g^ =Unemployed; ^h^ =Yes response; ^i^ =Fair; ^j^ =No response
