# Supplementary material for: Predictors of Smartphone and Tablet Use Among Patients With Hypertension: Secondary Analysis of Health Information National Trends Survey Data
Source: J Med Internet Res. 2022 Jan 24;24(1):e33188. doi: 10.2196/33188 (PMC8822436; doi:10.2196/33188)
Supplement: Multimedia Appendix 2 [file jmir_v24i1e33188_app2.docx]

(sample size = 3045; estimated population size = 183,285,150)

| Predictor and category | | Odds ratio (95% CI) | SE | *P*-value |
| --- | --- | --- | --- | --- |
|  |  |  |  |  |
| **Age group**^a^ **(years)** | | | | |
|  | 35-49 | 1.60 (0.646-3.960) | 0.463 | .32 |
|  | 50-64 | 1.04 (0.434-2.473) | 0.444 | .94 |
|  | 65-74 | 0.83 (0.324-2.106) | 0.477 | .69 |
|  | ≥75 | 0.60 (0.206-1.732) | 0.543 | .35 |
|  |  |  |  |  |
| **Gender**^b^ | | | | |
|  | Female | 1.22 (0.929-1.606) | 0.140 | .17 |
| **Education level**^c^ | | | | |
|  | High school graduate | 0.89 (0.417-1.895) | 0.386 | .76 |
|  | Some college | 1.28 (0.578-2.837) | 0.405 | .55 |
|  | College graduate or more | 1.17 (0.475-2.904) | 0.462 | .73 |
| **Race/ethnicity**^d^ | | | | |
|  | Non-Hispanic Black or African American | 0.94 (0.583-1.499) | 0.241 | .78 |
|  | Hispanic | 0.76 (0.421-1.371) | 0.301 | .37 |
|  | Non-Hispanic Asian | 0.86 (0.358-2.070) | 0.447 | .74 |
|  | Non-Hispanic other | 1.42 (0.503-4.010) | 0.530 | .52 |
| **Marital status**^e^ | | | | |
|  | Married | 1.54 (0.815-2.908) | 0.324 | .19 |
|  | Previously married | 1.31 (0.638-2.709) | 0.369 | .47 |
| **House-hold yearly income**^f^ **(US$)** | | | | |
|  | <20,000 | 0.26 (0.132-0.512) | 0.346 | ˂.001 |
|  | 20,000 to <35,000 | 0.35 (0.202-0.614) | 0.283 | .001 |
|  | 35,000 to <50,000 | 0.49 (0.276-0.881) | 0.296 | .03 |
|  | 50,000 to <75,000 | 0.60 (0.409-0.872) | 0.193 | .014 |
| **Employment status**^g^ | | | | |
|  | Employed | 0.81 (0.506-1.284) | 0.237 | .37 |
| **Smoked at least 100 cigarettes**^h^ | | | | |
|  | No | 1.12 (0.858-1.456) | 0.135 | .42 |
| **Health status**^i^ | | | | |
|  | Very good | 1.26 (0.660-2.087) | 0.241 | .35 |
|  | Good | 1.17 (0.789-2.087) | 0.226 | .49 |
| **BMI** | | | | |
|  |  | 1.01 (0.984-1.035) | 0.013 | .47 |
| **Diabetes**^j^ | | | | |
|  | Yes | 1.16 (0.852-1.585) | 0.158 | .35 |
| **Heart condition**^j^ | | | | |
|  | Yes | 1.09 (0.743-1.585) | 0.193 | .68 |
| **Depression**^j^ | | | | |
|  | Yes | 1.29 (0.816-2.036) | 0.233 | .29 |

^a-j^Reference categories for categorical predictors.

^a^ =18 to 34 years; ^b^ =Male; ^c^ =Less than high school; ^d^ =Non-Hispanic White; ^e^ =Never married; ^f^ = ≥US$75,000; ^g^ =Unemployed; ^h^ =Yes response; ^i^ =Fair; ^j^ =No response
